# Supplementary material for: The interactome of intact mitochondria by cross-linking mass spectrometry provides evidence for coexisting respiratory supercomplexes
Source: Mol Cell Proteomics. 2017 Dec 8;17(2):216–32. doi: 10.1074/mcp.RA117.000470 (PMC5795388; doi:10.1074/mcp.RA117.000470)
Supplement: Supplemental Data [file supp_RA117.000470_133922_0_supp_23978_fzffwf.zip › spectra_annotation/mito_spectra_annotation/17-1-30-1-6-1.pdf]

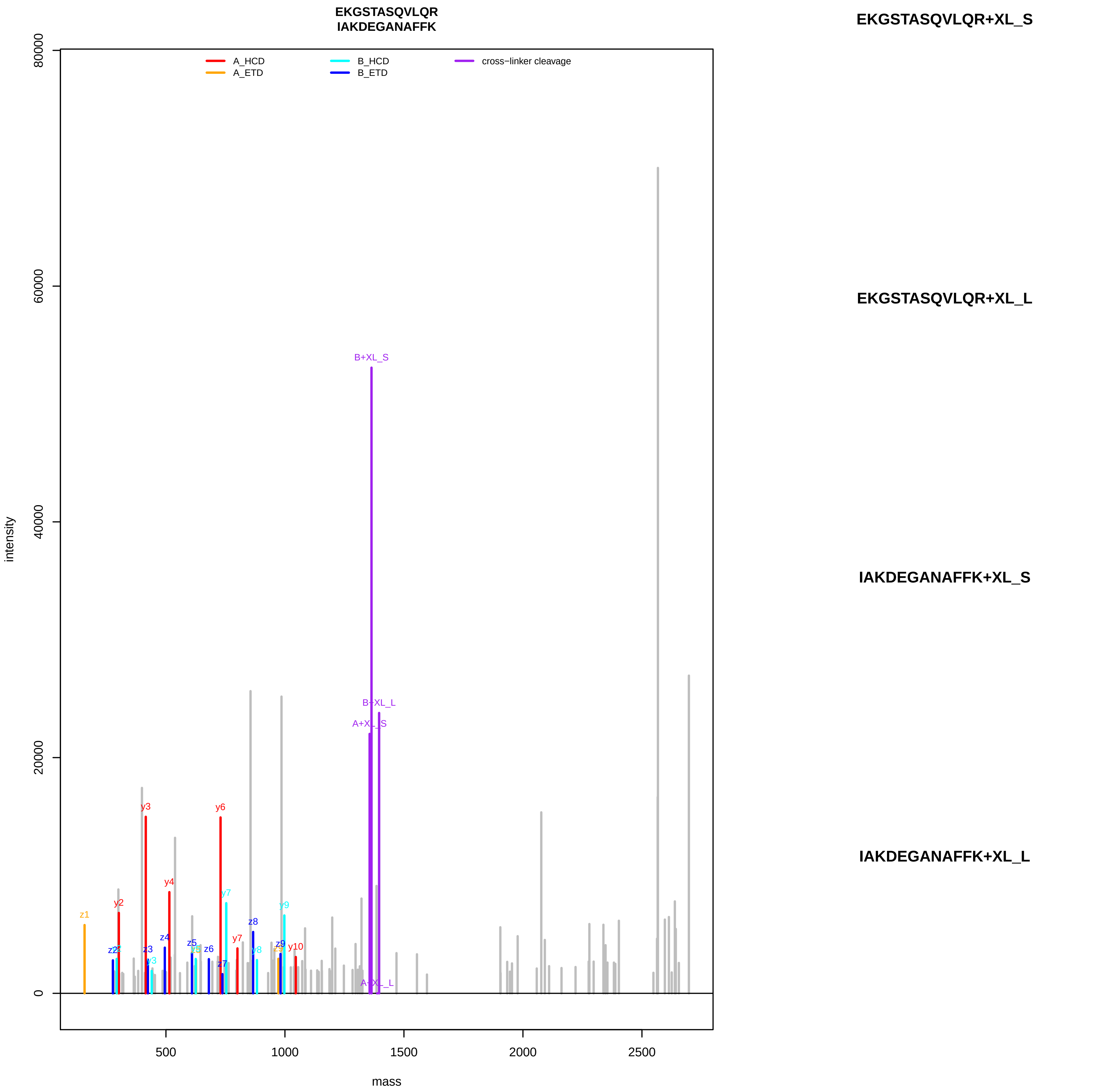
EKGSTASQVLQR
IAKDEGANAFFK
A_HCD
A_ETD
B_HCD
B_ETD
cross−linker cleavage
intensity
mass
0
20000
40000
60000
80000
500
1000
1500
2000
2500
B+XL_S
B+XL_L
A+XL_S
A+XL_L
z1
y2
z2
y3
z3
y3
y4
z4
z5
y5
z6
y6
y7
z7
y7
z8
y8
z9
y9
y10
EKGSTASQVLQR+XL_S
EKGSTASQVLQR+XL_L
IAKDEGANAFFK+XL_S
IAKDEGANAFFK+XL_L
